# Molecular Docking and Drug-Likeness of *Salicornia*-Derived Phytochemicals Against HER Receptors

**DOI:** 10.3390/cimb47070495

**Published:** 2025-06-27

**Authors:** Thiwanga N. Withana, Dinum Perera, Tharani D. Fernando

**Affiliations:** Department of Bioprocess Technology, Faculty of Technology, Rajarata University of Sri Lanka, Mihintale 50300, Sri Lanka; nirmanithiwanga@gmail.com

**Keywords:** epidermal growth factor receptor, in silico, virtual screening, ADME-Tox, anti-cancer therapy, halophyte

## Abstract

Cancer remains a major global public health concern, driving the need for innovative therapeutic agents with intensified efficacy and safety. Growth factor receptors (GFRs), often overexpressed in cancer cells and critical in regulating cell proliferation, survival, and tumor progression, represent key targets for cancer therapy. Halophytic plants like *Salicornia* spp. are known for their diverse bioactive compounds with notable pharmacological properties. This study comprehensively evaluated the anti-cancer potentials of phytochemicals derived from *Salicornia herbacea* and *Salicornia brachiata* using molecular docking and ADME-Tox (absorption, distribution, metabolism, excretion, and toxicity) profiling. A total of 37 bioactive compounds from *Salicornia* spp. were screened against HER1, HER2, and HER4 receptors. Among them, 3,5-di-O-caffeoylquinic acid, 3-O-caffeoylquinic acid, myricetin, quercetin, stigmasterol, kaempferol, isorhamnetin, rhamnetin, and hesperitin featured strong predicted binding affinities to the HER1, HER2, and HER4 growth factor receptors, comparable to those of standard anti-cancer drugs such as gefitinib and dovitinib. Further pharmacokinetic assessments, including bioavailability and toxicity analyses, identified compounds with favorable drug-likeness properties and minimal toxicity risks, except for myricetin and quercetin. These findings underscore the potential of *Salicornia*-derived phytochemicals as promising candidates for the development of safe, novel, and effective anti-cancer agents targeting GFRs, contributing to the advances in precision oncology, pending further validation through in vitro and/or in vivo experiments.

## 1. Introduction

Cancer remains a significant global health threat, with millions of new cases reported annually, contributing to high mortality rates despite advancements in chemotherapy, radiotherapy, immunotherapy, and surgeries [1,2,3,4]. These conventional cancer treatments often come with several drawbacks, including limited specificity, adverse side effects, non-specific toxicity, and the development of drug resistance, which hinder long-term treatment efficacy [5,6]. These challenges have sparked a growing interest in developing more effective anti-cancer agents, particularly those derived from safer natural products. Such agents are valued for their bioavailability, structural diversity, and lower toxicity than synthetic drugs [7,8,9].

Amongst natural sources, halophytic plants, such as *Salicornia herbacea* and *Salicornia brachiata*, which belong to the Amaranthaceae family [10], have been recognized for their unique bioactive compounds that exhibit a range of pharmacological potentials [8,11,12]. *S. herbacea* and *S. brachiata* are prominent halophytes that thrive in saline environments and have been traditionally used in East Asian medicine to treat disorders such as obesity, constipation, diabetes, and cancer [11,13].

Researchers have identified numerous bioactive phytochemical compounds, including flavonoids, phenolics, and saponins within *Salicornia* spp., which exhibit various therapeutic properties, such as antioxidants, anti-cancer, and anti-inflammatory activities [11,13,14]. Several in vitro and in vivo studies, as well as clinical trials, have been conducted to evaluate the therapeutic potential of these phytochemicals derived from different *Salicornia* spp. for treating various disease conditions, including memory dysfunction, breast cancer, obesity, and ischemic attacks [15,16,17]. Moreover, previous studies on the anti-cancer effects of *Salicornia* have shown that these phytochemicals interfere with specific molecular pathways involved in cancer cell proliferation, including apoptosis, cell cycle regulation, and metastasis, suggesting their potential as a novel strategy for cancer treatment [18,19,20,21].

When *Salicornia*-derived compounds are considered as potential candidates for anti-cancer drug discovery, in silico approaches such as molecular docking and ADME-Tox profiling play a vital role in the early stages of development. These methods help narrow down the list of bioactive compounds, which can then be further validated through in vitro and in vivo experiments. Molecular docking, which estimates the binding affinities between cancer-related proteins and bioactive molecules, aids in identifying potential inhibitors by elucidating molecular interactions and their inhibitory potential [22,23]. This approach saves both time and cost by prioritizing promising drug candidates before proceeding to laboratory testing. Complementing this, ADME-Tox profiling facilitates the evaluation of metabolic bioavailability, stability, and potential toxicity of the compounds [24]. By enabling early detection of pharmacokinetic and safety issues, it helps avoid investing resources in unviable candidates. These integrated in silico approaches are invaluable in the initial development of receptor-targeted therapeutic drug delivery systems for cancer and represent a significant advancement in early-stage drug discovery efforts [25,26].

The findings from this study underscore the potential of *S. herbacea* and *S. brachiata* phytochemicals as promising anti-cancer agents targeting HER family receptors, thereby providing a foundation for future in vitro and in vivo validation studies and further development of receptor-targeted therapies.

## 2. Materials and Methods

### 2.1. Retrieval and Preparation of Proteins

The kinase domains of human endothelial receptors HER1, HER2, and HER4 were selected for this study. The 3D crystal structures of these receptors (HER1: ID 2ITW [27]; HER2: ID 3PPO [28]; HER4: ID 2R4B [29]) were downloaded from the RCSB protein data bank (http://www.rcsb.org, accessed on 12 December 2024). The protein structures were then individually imported into the protein preparation module of AutoDockTools (version 1.5.7) [30]. Water molecules were removed, polar hydrogens were added, and Kollman charges were assigned. The prepared protein structures were then saved in PDBQT format for molecular docking analysis.

### 2.2. Ligand Selection and Preparation

A total of 37 bioactive compounds from *S. herbacea* and *S. brachiata*, known for their anti-cancer potential, were selected based on an extensive literature review of public databases and research articles. These bioactive compounds were downloaded from the PubChem (https://pubchem.ncbi.nlm.nih.gov, accessed on 12 December 2024) in SDF format, as 3D structures. Additionally, two standard anti-cancer drugs, gefitinib (CID-123631) and dovitinib (CID-135398510), were included for comparative analysis. All ligand structures were converted to PDB format using open babel software (http://openbabel.org, accessed on 15 December 2024). Subsequently, torsion parameters and docking configurations were refined using the AutoDock Tools (version 1.5.7) to optimize binding affinity and conformational flexibility.

### 2.3. Molecular Docking Analysis and Visualization

Molecular docking studies were performed by using AutoDock Vina (v1.1.2) [31] to evaluate the binding interactions between selected ligands and the target receptor proteins. A hybrid scoring function has been employed by AutoDock Vina, and it assesses the binding affinities based on empirical free energy. The binding affinity is expressed in kcal/mol, and more negative values indicate stronger interactions between the receptor and the ligand. It uses a combination of statistical potentials and empirical terms such as hydrogen bonding, hydrophobic interactions, conformational entropy, and geometric fit to estimate the most favorable ligand binding pose.

A 3D grid was generated around the active site of each receptor using AutoDockTools (version 1.5.7). The dimensions of the grid were set to 60 × 60 × 60 Å along the x, y, and z axes, with a resolution of 0.503 Å. The grid box was centered to encompass the binding pocket, ensuring accurate docking results. The grid configuration file was saved in txt format, and auto grid was executed with an exhaustiveness value of 50. Binding affinity values in (kcal/mol) were extracted from the log.txt file. The resulting ligand–protein interactions were visualized in UCSF ChimeraX (ChimeraX 1.9) [32] and Discovery Studio Visualizer (version 2024) [33] to interpret binding conformations.

The reliability of the molecular docking protocol was evaluated by redocking known control ligands, obtained from the DUD-E (Directory of Useful Decoys: Enhanced) database, into the HER1 receptor [34].

### 2.4. In Silico ADME Analysis

Lipinski’s Rule of Five (RO5) was implemented to investigate the drug-likeness, which serves as an indicator of oral bioavailability, of compounds that exhibited binding affinities comparable to those of standard drugs. According to Lipinski’s RO5, drug-likeness is evaluated based on five key criteria—molecular weight, number of hydrogen bond donors, number of hydrogen bond acceptors, lipophilicity (Log P), and molar refractivity—and compliance with these parameters was assessed using drug design software available at the supercomputing facility for bioinformatics and computational biology (SCFBio) at https://scfbio-iitd.res.in/software/drugdesign/lipinski.jsp (accessed on 20 December 2024) [35,36]. Additionally, the canonical SMILES of each ligand were obtained from PubChem and evaluated using SwissADME (http://www.swissadme.ch/index.php, accessed on 20 December 2024) [37] to predict key pharmacokinetic properties. These included gastrointestinal (GI) absorption, blood–brain barrier permeation (BBB), estimated solubility (ESOL), P-glycoprotein (P-gp) substrate status, and potential inhibition of major cytochrome P450 enzymes (such as CYP3A4 and CYP1A2), and bioavailability scores. These evaluations aimed to identify promising candidates for further development in the drug discovery pipeline.

### 2.5. Toxicity Analysis of Selected Compounds

Phytochemicals that exhibited both favorable binding affinities and compliance with Lipinski’s RO5 were further analyzed for toxicity profiles using the ProTox-III platform, as outlined by Banerjee et al. [38]. The toxicity assessment included the prediction of oral LD50 values, toxicity class, and model accuracy, as well as evaluations of potential hepatotoxicity, neurotoxicity, nephrotoxicity, respiratoxicity, and cardiotoxicity [38]. This analysis was essential for identifying compounds with minimal predicted toxicological risks, thereby supporting their potential as drug candidates.

## 3. Results and Discussion

### 3.1. Molecular Docking Analysis

**Figure 1:**
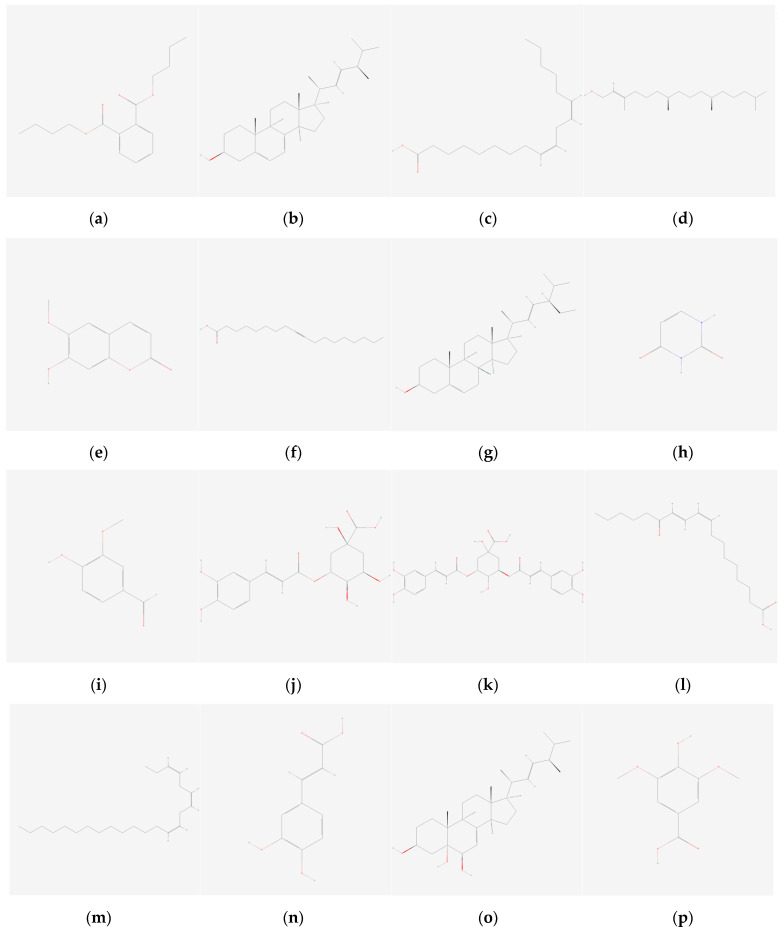

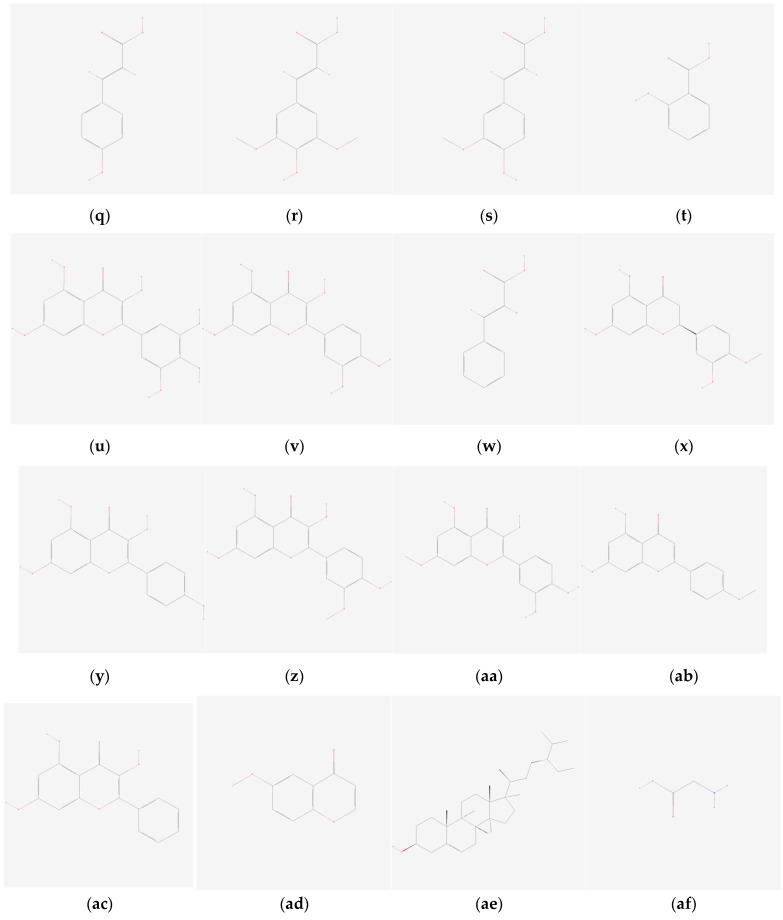

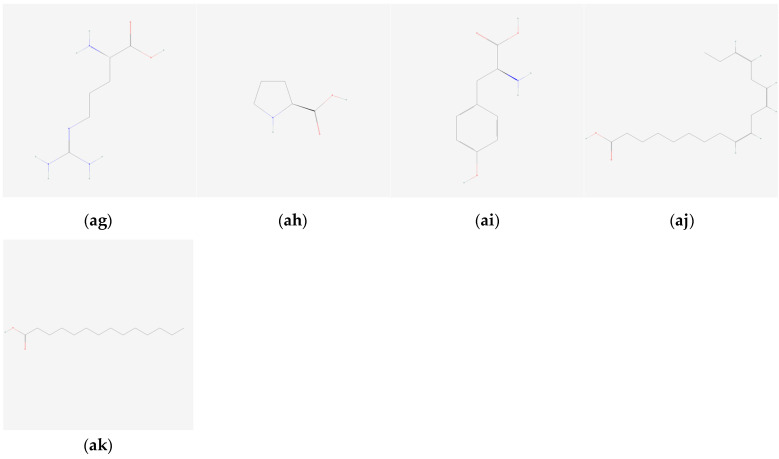
Two-dimensional structures of phytochemicals derived from *S. herbacea* and *S. brachiata* used in this study, along with their corresponding PubChem IDs: (**a**) dibutyl phthalate (CID: 3026); (**b**) ergosterol (CID: 444679); (**c**) linoleic (CID: 5280450); (**d**) phytol (CID: 5280435); (**e**) scopoletin (CID: 5280460); (**f**) stearolic (CID: 68167); (**g**) stigmasterol (CID: 5280794); (**h**) uracil (CID: 1174); (**i**) vanillic aldehyde (CID: 1183); (**j**) 3−O−caffeoylquinic acid (CID: 1794427); (**k**) 3,5−di−O−caffeoyl quinic acid (CID: 6474310); (**l**) (9Z,11E)−13-oxooctadeca−9,11-dienoic acid (13−KODE) (CID: 6446027); (**m**) (3Z,6Z,9Z)−tricosa−3,6,9−triene (CID: 56936133); (**n**) caffeic acid (CID: 689043); (**o**) cerevisterol (CID: 10181133); (**p**) syringic acid (CID: 10742); (**q**) p−coumaric acid (CID: 637542); (**r**) sinapic acid (CID: 637775); (**s**) ferulic acid (CID: 445858); (**t**) salicylic acid (CID: 338); (**u**) myricetin (CID: 5281672); (**v**) quercetin (CID: 5280343); (**w**) (trans−cinnamic) cinnamic acid (CID: 444539); (**x**) hesperetin (CID: 72281); (**y**) kaempferol (CID: 5280863); (**z**) isorhamnetin (CID: 5281654); (**aa**) rhamnetin (CID: 5281691); (**ab**) acacetin (CID: 5280442); (**ac**) galangin (CID: 5281616); (**ad**) 6−methoxychromanone (CID: 688880); (**ae**) β−sitosterol (CID: 222284); (**af**) glycine (CID: 750); (**ag**) arginine (CID: 6322); (**ah**) proline (CID: 145742); (**ai**) tyrosine (CID: 6057); (**aj**) linolenic acid (CID: 5280934); (**ak**) myristic acid (CID: 11005).

In this study, 37 bioactive compounds (Figure 1) were tested against growth factor receptors, HER1, HER2, and HER4. The most commonly used anti-cancer target drugs, such as gefitinib and dovitinib, known to interact with these receptors, were included as standard controls. Their binding affinities were used for comparative analysis. Among the 37 compounds, 31 were derived from *S. herbacea*, while limited data were available for *S. brachiata*.

**Figure 2:**
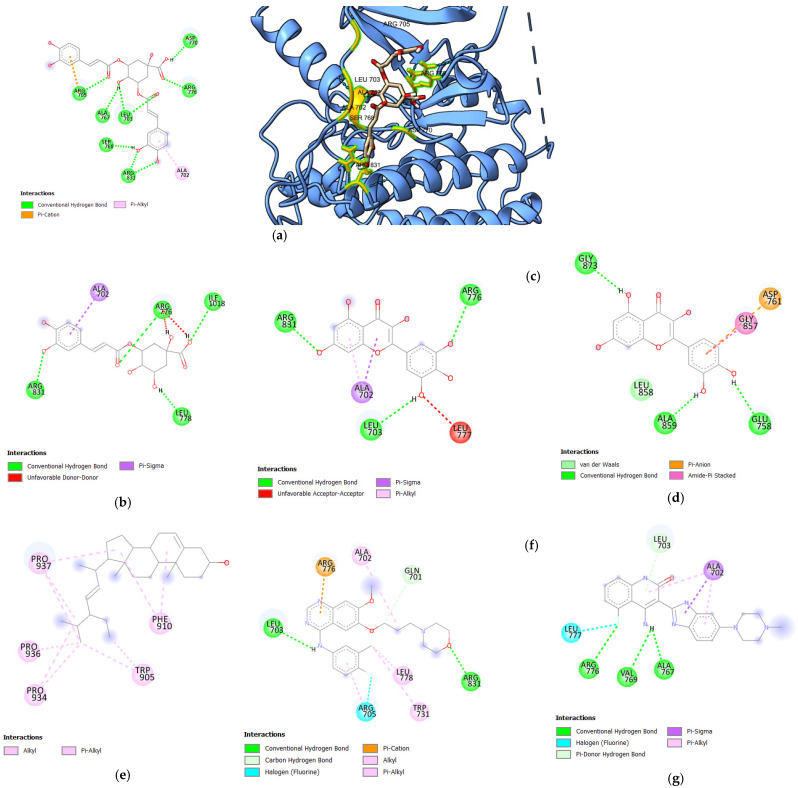
Diagrams illustrating the orientation of ligands within the binding pocket of HER1: (**a**) 2D interaction diagram (**left**) and 3D structural representation (**right**) of 3,5-di-O-caffeoylquinic acid; 2D interaction diagrams for (**b**) 3-O-caffeoylquinic acid, (**c**) myricetin, (**d**) quercetin, (**e**) stigmasterol, (**f**) gefitinib, and (**g**) dovitinib.

**Figure 3:**
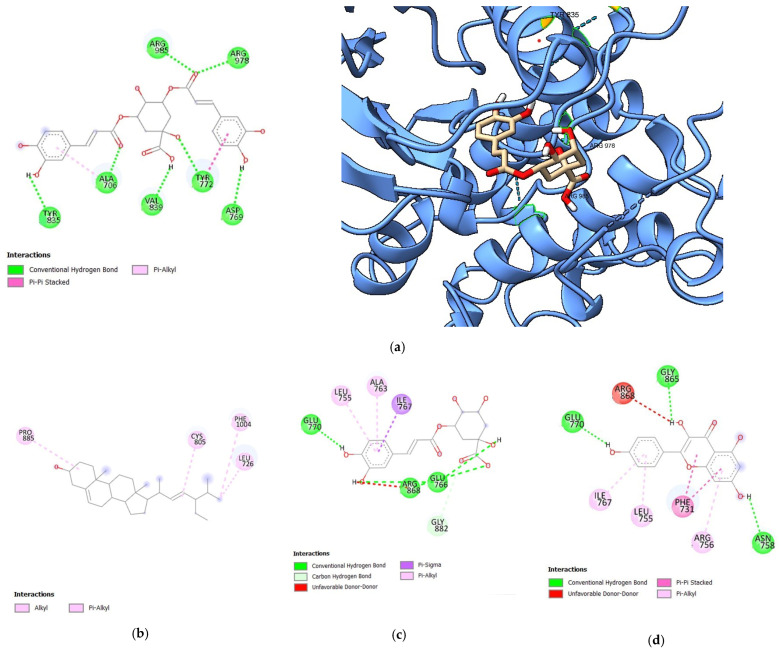

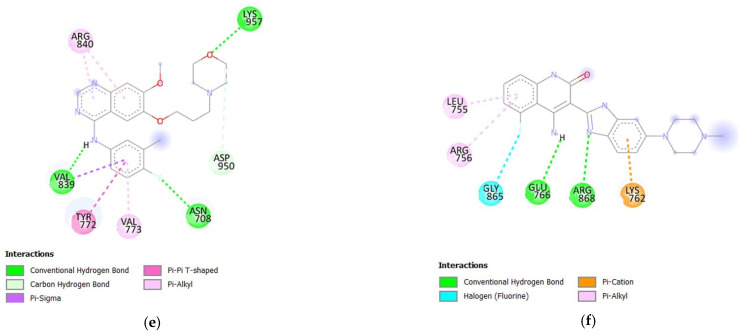
Diagrams illustrating the orientation of ligands within the binding pocket of HER2: (**a**) 2D interaction diagrams (**left**) and 3D structural representations (**right**) of 3,5-di-O-caffeoylquinic acid; 2D interaction diagrams for (**b**) stigmasterol, (**c**) 3-O-caffeoylquinic acid, (**d**) kaempferol, (**e**) gefitinib, and (**f**) dovitinib.

**Figure 4:**
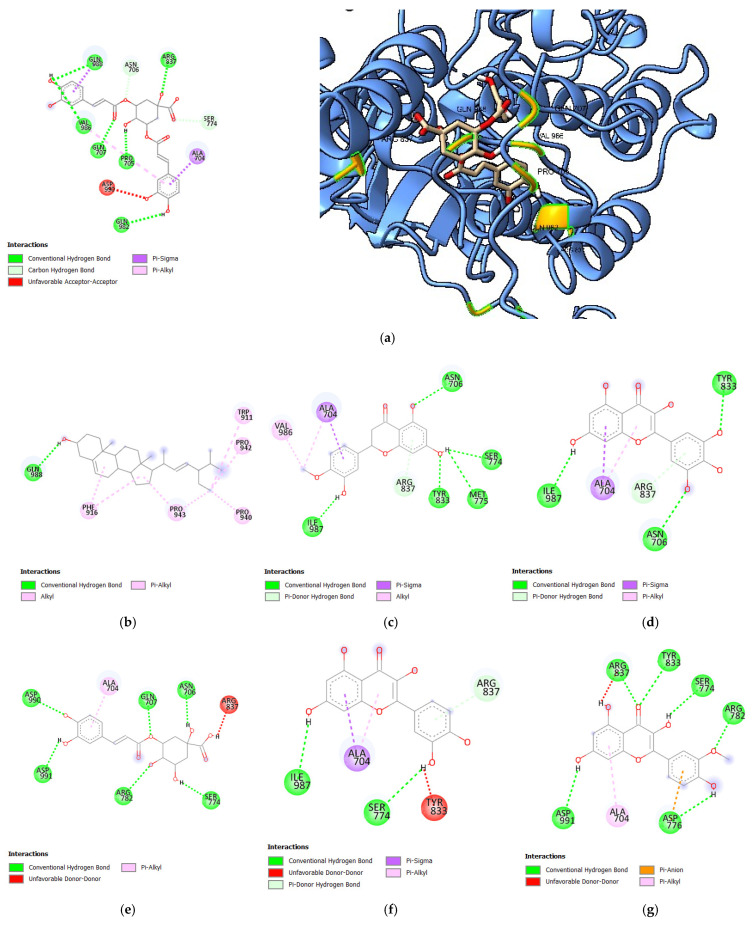

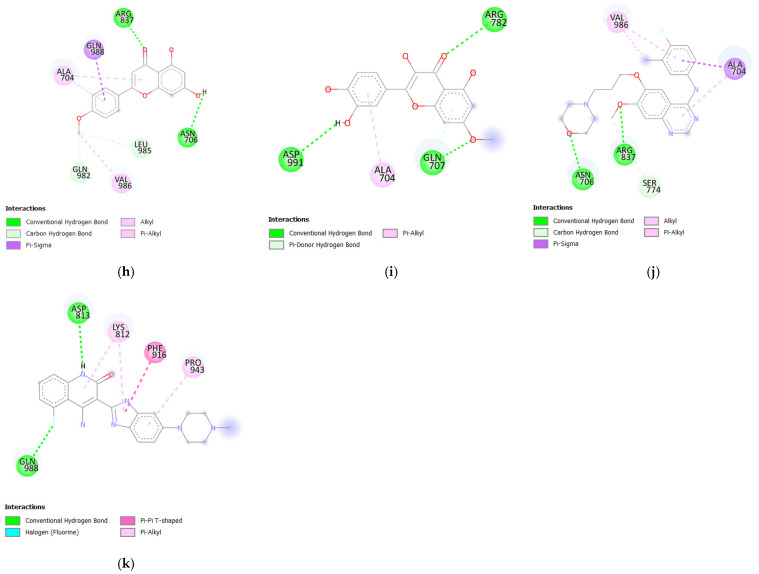
Diagrams illustrating the orientation of ligands within the binding pocket of HER4: (**a**) 2D interaction diagrams (**left**) and 3D structural representations (**right**) of HER4 3,5-di-O-caffeoylquinic acid; 2D interaction diagrams for (**b**) stigmasterol, (**c**) hesperetin, (**d**) myricetin, (**e**) 3-O-caffeoylquinic acid, (**f**) quercetin, (**g**) isorhamnetin, (**h**) acacetin, (**i**) rhamnetin, (**j**) gefitinib, and (**k**) dovitinib.

Following the docking, key parameters such as the binding affinity (kcal/mol), the number of hydrogen bonds, and the amino acids involved in hydrogen bonding were recorded. A validated docking procedure was employed to assess the efficacy of the bioactive compounds as potential inhibitors of HER1, HER2, and HER4 through in silico techniques. The binding affinity values of bioactive compounds from *S. herbacea* are presented in Table 1, Table 2 and Table 3, corresponding to HER1, HER2, and HER4, expressed as Gibbs free energy (kcal/mol). The bioactive compounds presented in these tables exhibited higher binding affinities than the standard drugs. Only the 3D structural representations of the highest-affinity ligands for each receptor are shown in Figure 2, Figure 3 and Figure 4, while the remaining interactions are included in Supplementary Figures S1–S3.

#### 3.1.1. Molecular Docking Analysis of Bioactive Compounds from *S. herbacea* and *S. brachiata* with HER1 Receptor

Docking analysis against the HER1 (Table 1) receptor revealed stronger binding affinities of 3,5-di-O-caffeoylquinic acid, 3-O-caffeoylquinic acid, myricetin, quercetin, and stigmasterol compared to the two standard drugs, gefitinib and dovitinib. Among the bioactive components in *S. herbacea*, 3,5-di-O-caffeoylquinic acid exhibited the highest binding affinity, with a binding energy of −8.7 kcal/mol, surpassing both standard drugs. Even though the binding affinities of 3-O-caffeoylquinic acid (−7.7 kcal/mol), myricetin (−7.6 kcal/mol), quercetin (−7.5 kcal/mol), and stigmasterol (−7.5 kcal/mol) against HER1 are slightly lower than the binding affinity of dovitinib, they were higher than that of gefitinib, indicating their high potential to interact with HER1. In contrast, the docking results for *S. brachiata* compounds against HER1 showed lower binding affinities than the standard drugs. The binding energies were recorded as 5.4, −4.4, −4.9, −4.2, −5.3, and 5.4 kcal/mol for arginine, glycine, linolenic acid, myristic acid, proline, and tyrosine, respectively.

The hydrogen bonding interactions of the standard drug dovitinib with HER1 involve three residues: ARG776, VAL769, and ALA767. Notably, dovitinib shared common interactions with the bioactive compounds 3,5-di-O-caffeoylquinic acid, 3-O-caffeoylquinic acid, and myricetin at the residues ALA767, ARG776, and ARG776, respectively. In contrast, gefitinib forms two hydrogen bonding interactions with HER1 at LEU703 and ARG831. Interestingly, the bioactive compounds 3,5-di-O-caffeoylquinic acid and myricetin also exhibited common interactions with HER1 at LEU703 and ARG831, similar to gefitinib. Additionally, 3-O-caffeoylquinic acid shares a common interaction with gefitinib at ARG831. When comparing the hydrogen bonding interactions of these bioactive compounds with HER1, 3,5-di-O-caffeoylquinic acid, 3-O-caffeoylquinic acid, and myricetin exhibit similar binding patterns to those of the standard drugs, dovitinib and gefitinib. Notably, 3,5-di-O-caffeoylquinic acid forms seven (07) hydrogen bonds compared to only three (03) for gefitinib and two (02) for dovitinib. A higher number of hydrogen bonds often correlates with enhanced receptor stability. Additionally, 3,5-di-O-caffeoylquinic acid interacts with a wider range of amino acids, including ASP770, ARG767, LEU703, ALA766, ARG705, ALA767, SER768, and ARG831, thereby strengthening the binding and accommodating variations in receptor conformation. Considering the binding affinities and the interactions of 3,5-di-O-caffeoylquinic acid, 3-O-caffeoylquinic acid, and myricetin, these compounds can be considered potential inhibitors of HER1, warranting further investigation.

The binding affinities of proposed compounds were compared with known active and inactive ligands obtained from DUD-E: Database (Table S1). According to the docking results, active compounds such as CID_5328245 and CID_2428 showed strong binding affinities of −8.5 and −8.1 kcal/mol, respectively, while inactive compounds like CID_135431661 and CID_5328753 exhibited weaker binding scores of −7.6 and −6.9 kcal/mol, respectively. Among the tested compounds, CID_6474310 (3,5-di-O-caffeoylquinic acid) demonstrated the highest binding affinity at −8.7 kcal/mol, surpassing several known active and inactive ligands and indicating a strong binding potential. In contrast, CID_1794427 (3-O-caffeoylquinic acid), the second proposed compound, achieved a binding score of −7.7 kcal/mol, placing it between the active and inactive reference compounds. These results support the reliability of the docking protocol for predictive screening.

#### 3.1.2. Molecular Docking Analysis of Bioactive Compounds from *S. herbacea* and *S. brachiata* with the HER2 Receptor

The binding affinities of the HER2 receptor (Table 2) with gefitinib and dovitinib were determined with respective binding energies of −7.8 kcal/mol and −9 kcal/mol. Among the bioactive compounds considered in this study, none exhibited a higher binding affinity than dovitinib (−9 kcal/mol). However, the binding affinities of 3,5-di-O-caffeoylquinic acid (−8.5 kcal/mol to −7.8 kcal/mol), stigmasterol (−8.1 kcal/mol), and 3-O-caffeoylquinic acid (−8 kcal/mol) were higher than that of gefitinib (−7.8 kcal/mol). Additionally, the bioactive compound kaempferol (−7.8 kcal/mol) in *S. herbacea* showed the same binding affinity as gefitinib (−7.8 kcal/mol).

3,5-di-O-caffeoylquinic acid formed strong interactions with the HER2 receptor, similar to its interactions with the HER1 receptor, involving seven (07) hydrogen bonds. Notably, 3,5-di-O-caffeoylquinic acid exhibited a similar interaction with the VAL839 residue in HER2, as observed with gefitinib. Although the binding affinity of stigmasterol is higher than that of gefitinib, it did not form any hydrogen bonds, indicating a weaker interaction. The bioactive compound 3-O-caffeoylquinic acid shared common interactions with HER2 at LARG868 and GLU766, similar to dovitinib. In contrast, all studied bioactive compounds from *S. brachiata*—arginine (−6.3 kcal/mol), glycine (−4.1 kcal/mol), linolenic acid (−6.0 kcal/mol), myristic acid (−5.3 kcal/mol), proline (−5.5 kcal/mol), and tyrosine (−6.6 kcal/mol)—exhibited weaker binding affinities than both gefitinib and dovitinib, indicating lower interaction strength with the HER2 receptor.

#### 3.1.3. Molecular Docking Analysis of Bioactive Compounds from *S. herbacea* and *S. brachiata* with the HER4 Receptor

When comparing the binding affinities of bioactive compounds from *S. herbacea* (Table 3) with the standard drugs, gefitinib and dovitinib against the HER4 receptor, all bioactive compounds exhibited lower binding affinities than dovitinib (−8.5 kcal/mol). However, common hydrogen bonding interactions were observed for 3,5-di-O-caffeoylquinic acid and stigmasterol at the amino acid residue GLN988. The binding affinities of the bioactive compounds 3,5-di-O-caffeoylquinic acid (−8.3 kcal/mol), stigmasterol (−7.9 kcal/mol), hesperetin (−7.7 kcal/mol), myricetin (−7.6 kcal/mol), 3-O-caffeoylquinic acid (−7.5 kcal/mol), quercetin (−7.5 kcal/mol), isorhamnetin (−7.4 kcal/mol), acacetin (−7.3 kcal/mol), and rhamnetin (−7.3 kcal/mol) were higher than that of gefitinib (−7.2 kcal/mol). In contrast, the studied compounds from *S. brachiata* demonstrated lower binding affinities with the HER4 receptor, suggesting weaker interaction capabilities.

Acacetin formed similar hydrogen bond interactions with the HER4 receptor at amino acid residues ASN706 and ARG837, comparable to gefitinib. Additionally, hesperetin, 3-O-caffeoylquinic acid, and isorhamnetin exhibited common hydrogen bond interactions with the HER4 receptor at amino acid residues ASN706, ASN706, and ARG837, respectively, as observed with gefitinib. These findings support the potential of these compounds as HER4-targeted inhibitors.

### 3.2. Drug-Likeness Properties of the Selected Phytochemicals According to Lipinski’s Rule of Five

Lipinski’s RO5 is commonly used to evaluate the drug-likeness of compounds, particularly for assessing their potential as orally active drugs. According to the rule, a compound is considered to have favorable drug-likeness if it does not violate more than one of the five criteria. According to the results, the quercetin, hesperitin, and rhamnetin satisfied all five criteria of Lipinski’s RO5, while 3,5-di-O-caffeoylquinic acid, myricetin, and stigmasterol each violated only one criterion, indicating favorable drug-likeness properties [39,40]. However, 3,5-di-O-caffeoylquinic acid and isorhamnetin violated Lipinski’s RO5, suggesting potential limitations in their drug-likeness. The detailed Lipinski’s RO5 parameter values for each phytochemical are presented in Table 4.

### 3.3. Pharmacokinetic Properties of Selected Phytochemicals According to SwissADME Analysis

The in silico pharmacokinetic analysis of the ligands, as shown in Table 5, indicates that the pharmacokinetic profiles and drug-likeness of several phytochemicals are comparable to those of standard drugs. ESOL-based solubility (ESOL) analysis revealed that the 3-O-caffeoylquinic acid, myricetin, quercetin, isorhamnetin, rhamnetin, and 3,5-di-O-caffeoylquinic acid exhibited ESOL values similar to those of dovitinib but lower than gefitinib.

Gastrointestinal absorption (GIA) and blood–brain barrier (BBB) penetration were predicted using the BOILED-Egg model, which considers the effects of polarity and lipophilicity on drug absorption [41]. Among the analyzed ligands, quercetin, hesperetin, and rhamnetin showed high GIA, while 3,5-di-O-caffeoylquinic acid, 3-O-caffeoylquinic acid, myricetin, isorhamnetin, and stigmasterol exhibited lower GIA values.

The bioavailability scores of most ligands—including myricetin, quercetin, hesperitin, stigmasterol, and rhamnetin—were within the normal range as commonly observed for many oral drugs. Moreover, a significant challenge in cancer treatment is the overexpression of P-glycoproteins (P-gp) in cancer cells, which promotes drug efflux and reduces the efficacy of chemotherapy. Being a substrate for P-gp can lead to decreased absorption, limited permeability, reduced oral bioavailability, and shortened drug retention time within the gastrointestinal tract [42]. In this study, the phytochemicals 3-O-caffeoylquinic acid, myricetin, quercetin, stigmasterol, rhamnetin, and gefitinib were found not to be substrates for P-gp, suggesting their potential for greater effectiveness in cancer treatment.

Phytochemicals such as 3,5-di-O-caffeoylquinic acid, 3-O-caffeoylquinic acid, isorhamnetin, and stigmasterol do not interfere with CYP3A4 and CYP1A2 isoenzymes, as shown in Table 5. These enzymes are key members of the cytochrome P450 (CYP) superfamily, which plays a crucial role in the biotransformation of drugs. Interactions of potential drug compounds with CYP isoenzymes can lead to two undesirable outcomes: enzyme induction, where the compound becomes a substrate and enhances enzyme activity, resulting in increased metabolism and reduced drug efficacy, or enzyme inhibition, where the compound interferes with enzyme function, causing drug accumulation and potential toxicity. Both scenarios can significantly impact drug safety and therapeutic outcomes [43,44].

### 3.4. Toxicity Analysis

**Figure 5:**
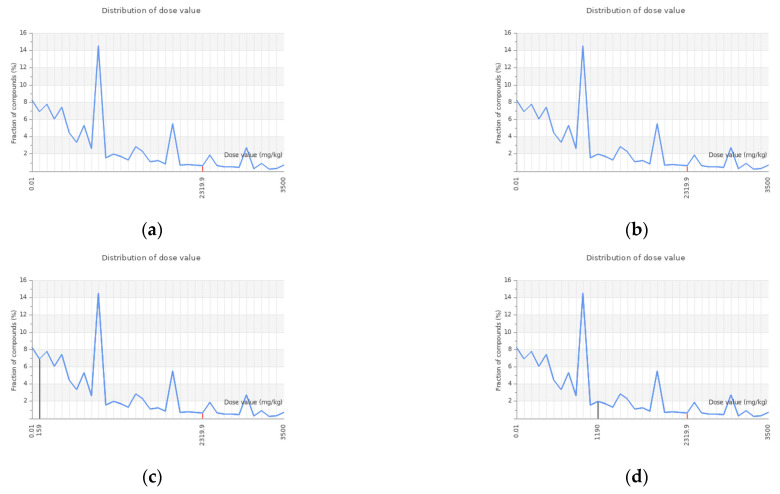

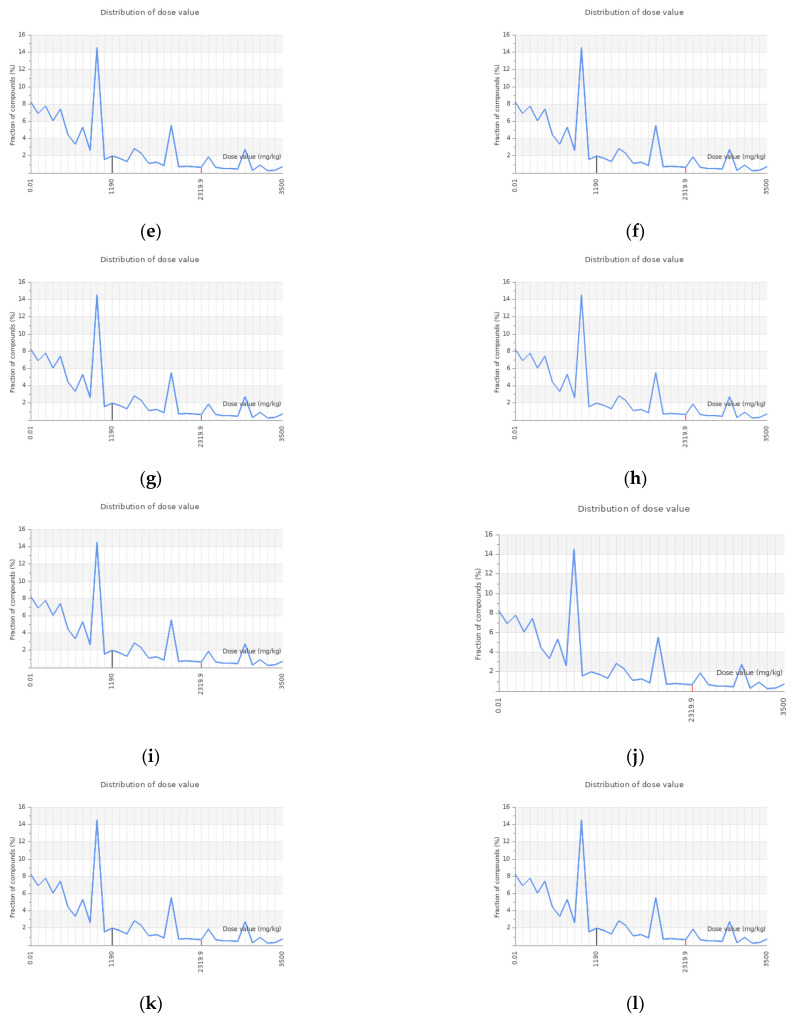
Graphical representation of predicted dose value distribution for studied compounds using the ProTox-III platform: (**a**) 3,5-di-O-caffeoylquinic acid; (**b**) 3-O-caffeoylquinic acid; (**c**) myricetin; (**d**) quercetin; (**e**) stigmasterol; (**f**) kaempferol; (**g**) isorhamnetin; (**h**) rhamnetin); (**i**) hesperitin; (**j**) acacetin; (**k**) gefitinib); (**l**) dovitinib.

Table 6 presents the oral acute toxicity (LD50 by mg/kg), prediction accuracy, toxicity classification, and average similarity. Among the tested phytochemicals, myricetin and quercetin exhibited the highest oral toxicity, with an LD50 value of 159 mg/kg, placing them in toxicity class 3 (50 < LD50 ≤ 300 mg/kg) (Figure 5). Compounds in this class are considered highly toxic, with a strong potential to cause adverse effects upon ingestion, as indicated by 100% prediction accuracy and high structural similarity. In contrast, the oral toxicity values for 3,5-di-O-caffeoylquinic acid, 3-O-caffeoylquinic acid, kaempferol, isorhamnetin, rhamnetin, and acacetin were 5000 mg/kg, 5000 mg/kg, 3919 mg/kg, 5000 mg/kg, 5000 mg/kg, and 4000 mg/kg, respectively. These phytochemicals, along with the standard drug gefitinib, fall under toxicity class 5 (2000 < LD50 ≤ 5000), indicating a relatively low toxicity profile. Furthermore, stigmasterol and hesperitin belong to toxicity class 4 (300 < LD50 ≤ 2000 mg/kg), the same classification as the standard anti-cancer drug dovitinib. Since phytochemicals classified under classes 4 and 5 are generally considered safer, they hold significant potential for further drug development due to their lower toxicity levels [45,46].

### 3.5. Prediction of Organ-Specific Toxicity of the Selected Compounds in S. herbacea

Furthermore, organ-specific toxicity analyses were carried out, and the results are presented in Table 7. The hepatotoxicity assessment indicated that all of the selected bioactive compounds from *S. herbacea* were predicted to be inactive, suggesting a lower risk of liver toxicity. Similarly, neurotoxicity was predicted to be inactive for all compounds except for stigmasterol. In contrast, both standard drugs, gefitinib and dovitinib, exhibited both hepatotoxicity and neurotoxicity activity, highlighting potential adverse effects associated with their use.

Regarding nephrotoxicity, all tested bioactive compounds showed activity, whereas gefitinib and dovitinib were inactive, suggesting a possible renal safety concern for the natural compounds. A similar trend was observed for cardiotoxicity, where both standard drugs were inactive, while isorhamnetin, hesperetin, and acacetin exhibited cardiotoxic activity among the bioactive compounds.

Additionally, all phytochemicals, as well as the standard drugs, exhibited respiratory toxicity, indicating that these compounds should not be administered via inhalation. Given these findings, further in vivo and in vitro experimental validation is essential to accurately assess the safety and pharmacological potential of these compounds in future studies.

## 4. Conclusions

This study highlights the potential of *Salicornia*-derived phytochemicals as promising candidates for targeted cancer therapy through the inhibition of human epidermal growth factor receptors (HER1, HER2, and HER4). By employing virtual screening methods—including molecular docking and ADME-Tox profiling—a total of 37 bioactive compounds from *Salicornia herbacea* (31) and *Salicornia brachiata* (06) were evaluated for their anti-cancer potential.

Among the screened compounds, phytochemicals derived from *Salicornia herbacea*—including 3,5-di-O-caffeoylquinic acid, 3-O-caffeoylquinic acid, myricetin, quercetin, stigmasterol, kaempferol, isorhamnetin, rhamnetin, hesperetin, and acacetin—demonstrated strong binding affinities to the HER receptors, comparable to standard anti-cancer agents such as gefitinib and dovitinib. These top-performing phytochemicals also exhibited favorable drug-likeness and pharmacokinetic profiles, supporting their viability as oral drug candidates. Although most compounds were predicted to have low toxicity risks, myricetin and quercetin showed potential concerns, warranting careful consideration in future studies.

The findings highlight the significance of *Salicornia* as a promising source of novel anti-cancer compounds, offering new prospects for applications in precision oncology. However, further in vitro and in vivo validation is essential to confirm the efficacy, safety, and mechanism of action of these compounds before clinical translation.

## Figures and Tables

**Table 1:** Binding parameters of bioactive compounds from *S. herbacea* against the HER1 target protein.

Phytochemical	Binding Energy (ΔG) (kcal/mol)	Number of H Bonds	Amino Acids Involved in Hydrogen Bonding
3,5-di-O-caffeoylquinic acid	−8.7	7	ASP770, ARG767, LEU703, SER768, ARG831, ALA767, ALA766, ARG705
3-O-caffeoylquinic acid	−7.7	4	ARG831, ARG776, ILE1018, LEU778
Myricetin	−7.6	3	ARG831, ARG776, LEU703
Quercetin	−7.5	3	GLY873, ALA859, GLU758
Stigmasterol	−7.5	0	-
Gefitinib	−7.4	2	LEU703, ARG831
Dovitinib	−8.1	3	ARG776, VAL769, ALA767

**Table 2:** Binding parameters of *S. herbacea* bioactive compounds against the HER2 target protein.

Phytochemical	Binding Energy (ΔG) (kcal/mol)	Number of H Bonds	Amino Acids Involved in Hydrogen Bonding
3,5-di-O-caffeoylquinic acid	−8.5	7	ARG98, ASP769, ARG978, TYR835, ALA706, VAL839, TYR772
Stigmasterol	−8.1	0	-
3-O-caffeoylquinic acid	−8.0	3	GLU770, ARG868, GLU766
Kaempferol	−7.8	3	GLU770, GLY865, ASN758
Gefitinib	−7.8	3	LYS 957, VAL839, ASN708
Dovitinib	−9.0	2	GLU766, ARG868

**Table 3:** Binding parameters of bioactive compounds of *S. herbacea* against the HER4 target protein.

Phytochemical	Binding Energy (ΔG) (kcal/mol)	Number of H Bonds	Amino Acids Involved in Hydrogen Bonding
3,5-di-O-caffeoylquinic acid	−8.3	5	GLN988, ARG837, VAL986, GLN707, PRO705, GLN 982
Stigmasterol	−7.9	1	GLN988
Hesperitin	−7.7	5	ASN706, ILE987, TYR833, MET775, SER774
Myricetin	−7.6	3	ILE987, ASN706, TYR833
3-O-caffeoylquinic acid	−7.5	6	ASP990, ASP991, GLN707, ARG782, ASN706, SER774
Quercetin	−7.5	2	ILE987, SER774
Isorhamnetin	−7.4	6	ASP991, ARG837, TYR833, SER774, ARG782, ASP776
Acacetin	−7.3	2	ARG837, ASN706
Rhamnetin	−7.3	3	ARG782, ASP991, GLN707
Gefitinib	−7.2	2	ASN706, ARG837
Dovitinib	−8.5	2	ASP813, GLN988

**Table 4:** Drug-likeness properties of the selected phytochemicals based on Lipinski’s Rule of Five (RO5) criteria.

Phytochemical	Mass (g/mol)	Hydrogen Bond Donor	Hydrogen Bond Acceptor	Log P	Molar Refractivity
3,5-di-O-caffeoylqunic acid	516.45	7	12	0.81	126.90
Myricetin	318.24	6	8	1.69	80.06
Quercetin	302.24	5	7	1.99	78.03
Hesperitin	302.28	3	6	2.19	78.06
Isorhamnetin	478.40	7	12	−0.24	114.63
Stigmasterol	412.69	1	1	7.80	132.75
Rhamnetin	82.50	4	7	2.29	82.50
Kaempferol	286.24	4	6	1.90	76.01
Acacetin	284.26	2	5	3.35	78.46
Gefitinib	446.90	1	7	4.32	121.66
Dovitinib	392.43	3	4	2.21	120.28
3-O-caffeoylquinic acid	354.31	6	9	−0.75	83.50

**Table 5:** In silico pharmacokinetic properties of the studied ligands predicted using SwissADME.

Phytochemical	ESOL (Log S)	GIA	BBB Permeant	P-gp Substrate	CYP3A4 Inhibitor	CYPIA2 Inhibitors	Bioavailability Score
3,5-di-O-caffeoylquinic acid	−3.65	Low	No	Yes	No	No	0.11
3-O-caffeoylquinic acid	−1.62	Low	No	No	No	No	0.11
Myricetin	−3.01	Low	No	No	Yes	Yes	0.55
Quercetin	−3.16	High	No	No	Yes	Yes	0.55
Hesperitin	−3.62	High	No	Yes	Yes	Yes	0.55
Isorhamnetin	−3.26	Low	No	Yes	No	No	0.17
Stigmasterol	−7.46	Low	No	No	No	No	0.55
Rhamnetin	−3.36	High	No	No	Yes	Yes	0.55
Gefitinib	−5.05	High	Yes	No	Yes	No	0.56
Dovitinib	−3.66	High	No	Yes	No	Yes	0.55

ESOL = estimated solubility; GI = human gastrointestinal absorption; BBB = blood–brain barrier permeation.

**Table 6:** Predicted oral acute toxicity (LD50), toxicity class, and model accuracy for the studied compounds.

Compounds Name	Oral LD50 Value (mg/Kg)	Predicted Toxicity Class	Prediction Accuracy (%)	Average Similarity (%)
3,5-di-O-caffeoylquinic acid	5000	5	71.63	69.26
3-O-caffeoylquinic acid	5000	5	71.21	69.26
Myricetin	159	3	100	100
Quercetin	159	3	100	100
Stigmasterol	890	4	89.38	70.97
Kaempferol	3919	5	82.46	70.97
Isorhamnetin	5000	5	87.48	70.97
Rhamnetin	5000	5	86.79	70.97
Hesperitin	2000	4	77.77	69.26
Acacetin	4000	5	83.54	70.97
Gefitinib	2935	5	51.88	67.38
Dovitinib	1072	4	53.59	67.38

**Table 7:** Prediction of hepatotoxicity, neurotoxicity, nephrotoxicity, respiratory toxicity, and cardiotoxicity of selected phytochemicals identified in *S. herbacea*.

Compounds Name	Hepatotoxicity	Neurotoxicity	Nephrotoxicity	Respirotoxicity	Cardiotoxicity
3,5-di-O-caffeoylquinic acid	0.70 I	0.87 I	0.52 A	0.52 A	0.88 I
3-O-caffeoylquinic acid	0.72 I	0.89 I	0.56 A	0.57 A	0.99 I
Myricetin	0.69 I	0.89 I	0.62 A	0.83 A	0.99 I
Quercetin	0.69 I	0.89 I	0.62 A	0.83 A	0.99 I
Stigmasterol	0.87 I	0.54 A	0.89 I	0.82 A	0.85 I
Kaempferol	0.68 I	0.89 I	0.62 A	0.83 A	0.91 I
Isorhamnetin	0.72 I	0.88 I	0.64 A	0.85 A	0.82 A
Rhamnetin	0.73 I	0.86 I	0.59 A	0.82 A	0.71 I
Hesperitin	0.70 I	0.87 I	0.67 A	0.86 A	0.99 A
Acacetin	0.72 I	0.84 I	0.63 A	0.78 A	0.64 A
Gefitinib	0.73 A	0.83 A	0.51 I	0.98 A	0.81 I
Dovitinib	0.54 A	0.96 A	0.75 I	0.98 A	0.87 I

I = inactive; A = active.

## Data Availability

All data are available within the manuscript and Supplementary Materials.

## Supplementary Materials

The following supporting information can be downloaded at: https://www.mdpi.com/article/10.3390/cimb47070495/s1.

## References

[1] Siegel R.L., Giaquinto A.N., Jemal A. (2024). Cancer Statistics, 2024. CA Cancer J. Clin..

[2] Arthur R.S., Kabat G.C., Kim M.Y., Wild R.A., Shadyab A.H., Wactawski-Wende J., Ho G.Y.F., Reeves K.W., Kuller L.H., Luo J. (2019). Metabolic Syndrome and Risk of Endometrial Cancer in Postmenopausal Women: A Prospective Study. Cancer Causes Control.

[3] Bauso L.V., La Fauci V., Munaò S., Bonfiglio D., Armeli A., Maimone N., Longo C., Calabrese G. (2024). Biological Activity of Natural and Synthetic Peptides as Anticancer Agents. Int. J. Mol. Sci..

[4] Singh A. (2024). Global Burden of Five Major Types of Gastrointestinal Cancer. Gastroenterol. Rev..

[5] Zhou J., Sun H., Wang Z., Cong W., Zeng M., Zhou W., Bie P., Liu L., Wen T., Kuang M. (2023). Guidelines for the Diagnosis and Treatment of Primary Liver Cancer (2022 Edition). Liver Cancer.

[6] Boța M., Vlaia L., Jîjie A.-R., Marcovici I., Crişan F., Oancea C., Dehelean C.A., Mateescu T., Moacă E.-A. (2024). Exploring Synergistic Interactions between Natural Compounds and Conventional Chemotherapeutic Drugs in Preclinical Models of Lung Cancer. Pharmaceuticals.

[7] Newman D.J. (2024). Non-Insulin-Based Drug Entities Used to Treat Diabetes Type 2 Disease (T2DM), Based on Natural Products from All Sources. J. Nat. Prod..

[8] Chantrill L.A., Nagrial A.M., Watson C., Johns A.L., Martyn-Smith M., Simpson S., Mead S., Jones M.D., Samra J.S., Gill A.J. (2015). Precision Medicine for Advanced Pancreas Cancer: The Individualized Molecular Pancreatic Cancer Therapy (IMPaCT) Trial. Clin. Cancer Res..

[9] Newman D.J., Cragg G.M. (2020). Natural Products as Sources of New Drugs over the Nearly Four Decades from 01/1981 to 09/2019. J. Nat. Prod..

[10] Siridewa K., De Silva W., Ratnayake R.M.C.S., Wijesundara S., Perera D., Attanayake R.N. (2025). Species Identification and Pollination Biology of an Economically Important True Halophyte, *Salicornia brachiata* Roxb. Aquat. Bot..

[11] Ekanayake S., Egodawatta C., Attanayake R.N., Perera D. (2023). From Salt Pan to Saucepan: *Salicornia*, a Halophytic Vegetable with an Array of Potential Health Benefits. Food Front..

[12] Kang S., Kim M.-R., Chiang M., Hong J. (2015). Evaluation and Comparison of Functional Properties of Freshwater-Cultivated Glasswort (*Salicornia herbacea* L.) with Naturally-Grown Glasswort. Food Sci. Biotechnol..

[13] Kim S., Lee E.-Y., Hillman P.F., Ko J., Yang I., Nam S.-J. (2021). Chemical Structure and Biological Activities of Secondary Metabolites from *Salicornia europaea* L. *Molecules*
**2021**, *26*, 2252. Molecules.

[14] Sánchez-Gavilán I., Ramírez E., de la Fuente V. (2021). Bioactive Compounds in *Salicornia patula* Duval-Jouve: A Mediterranean Edible Euhalophyte. Foods.

[15] Lee W.-J., Shin Y.-W., Kim D.-E., Kweon M.-H., Kim M. (2020). Effect of Desalted *Salicornia europaea* L. Ethanol Extract (PM-EE) on the Subjects Complaining Memory Dysfunction without Dementia: A 12-Week, Randomized, Double-Blind, Placebo-Controlled Clinical Trial. Sci. Rep..

[16] Nájar A.M., López Azcárate C., Domínguez Ruiz C., Núñez-Jurado D., de Torres R., López R., Camino-Moya M., Magni E., Montero-Ramirez E., Bocero A. (2024). Evaluating the Clinical Impact of a Polyphenol-Rich Extract from *Salicornia ramosissima* on Patients with Transient Ischemic Attack and Minor Stroke. Nutrients.

[17] On J.-Y., Kim S.-H., Kim J.-M., Park S., Kim K.-H., Lee C.-H., Kim S.-K. (2023). Effects of Fermented *Artemisia annua* L. and *Salicornia herbacea* L. on Inhibition of Obesity In Vitro and In Mice. Nutrients.

[18] Lim B., Lin Y., Navin N. (2020). Advancing Cancer Research and Medicine with Single-Cell Genomics. Cancer Cell.

[19] Ko Y.-C., Choi H.S., Kim J.-H., Kim S.-L., Yun B.-S., Lee D.-S. (2020). Coriolic Acid (13-(S)-Hydroxy-9Z, 11E-Octadecadienoic Acid) from Glasswort *(Salicornia herbacea* L.) Suppresses Breast Cancer Stem Cell through the Regulation of c-Myc. Molecules.

[20] Doi N., Togari H., Minagi K., Nakaoji K., Hamada K., Tatsuka M. (2020). Protective Effects of *Salicornia europaea* on UVB-Induced Misoriented Cell Divisions in Skin Epithelium. Cosmetics.

[21] Karadeniz F., Kim J.A., Ahn B.N., Kwon M.S., Kong C.S. (2014). Effect of *Salicornia herbacea* on Osteoblastogenesis and Adipogenesis *in Vitro*. Mar. Drugs.

[22] Ahmad W., Ansari M.A., Alsayari A., Almaghaslah D., Wahab S., Alomary M.N., Jamal Q.M.S., Khan F.A., Ali A., Alam P. (2022). In Vitro, Molecular Docking and In Silico ADME/Tox Studies of Emodin and Chrysophanol against Human Colorectal and Cervical Carcinoma. Pharmaceuticals.

[23] Islam M.A., Hossain M.S., Hasnat S., Shuvo M.H., Akter S., Maria M.A., Tahcin A., Hossain M.A., Hoque M.N. (2024). In-Silico Study Unveils Potential Phytocompounds in Andrographis Paniculata against E6 Protein of the High-Risk HPV-16 Subtype for Cervical Cancer Therapy. Sci. Rep..

[24] Chebbac K., Abchir O., Chalkha M., El Moussaoui A., El Barnossi A., Lafraxo S., Chtita S., Salamatullah A.M., Bourhia M., Dauelbait M. (2024). Phytochemical Analysis, Antimicrobial and Antioxidant Activities of Essential Oils of the Species *Artemisia Mesatlantica* Maire: *In Vitro* and *in Silico* Approaches. CyTA-J. Food.

[25] Amaya-Rodriguez C.A., Carvajal-Zamorano K., Bustos D., Alegría-Arcos M., Castillo K. (2024). A Journey from Molecule to Physiology and *in Silico* Tools for Drug Discovery Targeting the Transient Receptor Potential Vanilloid Type 1 (TRPV1) Channel. Front. Pharmacol..

[26] Brogi S., Ramalho T.C., Kuca K., Medina-Franco J.L., Valko M. (2020). Editorial: In Silico Methods for Drug Design and Discovery. Front. Chem..

[27] Yun C.-H., Boggon T.J., Li Y., Woo S., Greulich H., Meyerson M., Eck M.J. (2007). Crystal Structure of EGFR Kinase Domain in Complex with AFN941. Worldw. Protein Data Bank.

[28] Du Y., Shi W.W., He Y.X., Yang Y.H., Zhou C.Z., Chen Y. (2011). Structures of the Substrate-Binding Protein Provide Insights into the Multiple Compatible Solutes Binding Specificities of Bacillus Subtilis ABC Transporter OpuC. Worldw. Protein Data Bank.

[29] Wood E.R., Shewchuk L.M., Ellis B., Brignola P., Brashear R.L., Caferro T.R., Dickerson S.H., Dickson H.D., Donaldson K.H., Gaul M. (2008). 6-Ethynylthieno[3,2-d]- and 6-Ethynylthieno[2,3-d] Pyrimidin-4-Anilines as Tunable Covalent Modifiers of ErbB Kinases. Proc. Natl. Acad. Sci. USA.

[30] Sathish S., Devaraju P., Julius A., Sohn H., Madhavan T. (2024). Identification of Selective Inhibitors for Janus Kinase 1: An Integrated Drug Repurposing Strategy for Breast Cancer. Chem. Pap..

[31] Eberhardt J., Santos-Martins D., Tillack A.F., Forli S. (2021). AutoDock Vina 1.2.0: New Docking Methods, Expanded Force Field, and Python Bindings. J. Chem. Inf. Model..

[32] Meng E.C., Goddard T.D., Pettersen E.F., Couch G.S., Pearson Z.J., Morris J.H., Ferrin T.E. (2023). UCSF ChimeraX: Tools for structure building and analysis. Protein Sci..

[33] BIOVIA, Dassault Systèmes, Discovery Studio, Release 2024. San. Diego: Dassault Systèmes. https://www.3ds.com/products/biovia/discovery-studio.

[34] Mysinger M.M., Carchia M., Irwin J.J., Shoichet B.K. (2012). Directory of Useful Decoys, Enhanced (DUD-E): Better Ligands and Decoys for Better Benchmarking. J. Med. Chem..

[35] Jayaram B., Singh T., Mukherjee G., Mathur A., Shekhar S., Shekhar V. (2012). Sanjeevini: A Freely Accessible Web-Server for Target-Directed Lead Molecule Discovery. BMC Bioinform..

[36] Lipinski C.A. (2004). Lead- and Drug-like Compounds: The Rule-of-Five Revolution. Drug Discov. Today Technol..

[37] Daina A., Michielin O., Zoete V. (2017). SwissADME: A Free Web Tool to Evaluate Pharmacokinetics, Drug-Likeness and Medicinal Chemistry Friendliness of Small Molecules. Sci. Rep..

[38] Banerjee P., Eckert A.O., Schrey A.K., Preissner R. (2018). ProTox-II: A Webserver for the Prediction of Toxicity of Chemicals. Nucleic Acids Res..

[39] Başar Y., Yenigün S., Gül F., Ozen T., Demirtas İ., Alma M.H., Temel S. (2024). Phytochemical Profiling, Molecular Docking and ADMET Prediction of Crude Extract of Atriplex nitens schkuhr for the Screening of Antioxidant and Urease Inhibitory. Int. J. Chem. Technol..

[40] Lipinski C.A., Lombardo F., Dominy B.W., Feeney P.J. (2012). Experimental and Computational Approaches to Estimate Solubility and Permeability in Drug Discovery and Development Settings. Adv. Drug. Deliv. Rev..

[41] Degfie T., Endale M., Begna T., Jung C., Dekebo A. (2024). GC-MS Profiling and in Silico Pharmacokinetic Properties of Essential Oils Hydrodistilled from Leaves of Capparis Tomentosa and Cadaba Rotundifolia. Bull. Chem. Soc. Ethiop..

[42] Amin M.L. (2013). P-Glycoprotein Inhibition for Optimal Drug Delivery. Drug Target Insights.

[43] Antonovic L., Martinez M. (2011). Role of the Cytochrome P450 Enzyme System in Veterinary Pharmacokinetics: Where Are We Now? Where Are We Going?. Future Med. Chem..

[44] Lei Z., Tian Q., Teng Q., Wurpel J.N.D., Zeng L., Pan Y., Chen Z. (2023). Understanding and Targeting Resistance Mechanisms in Cancer. MedComm.

[45] Drwal M.N., Banerjee P., Dunkel M., Wettig M.R., Preissner R. (2014). ProTox: A Web Server for the *in-Silico* Prediction of Rodent Oral Toxicity. Nucleic Acids Res..

[46] Singh N., Vayer P., Tanwar S., Poyet J.-L., Tsaioun K., Villoutreix B.O. (2023). Drug Discovery and Development: Introduction to the General Public and Patient Groups. Front. Drug Discov..

