# Supplementary material for: Molecular Docking and Drug-Likeness of Salicornia-Derived Phytochemicals Against HER Receptors
Source: Curr Issues Mol Biol. 2025 Jun 27;47(7):495. doi: 10.3390/cimb47070495 (PMC12293734; doi:10.3390/cimb47070495)
Supplement: Supplementary file 1 [file cimb-47-00495-s001.zip › Supplementary Figures S1-S3.pdf]

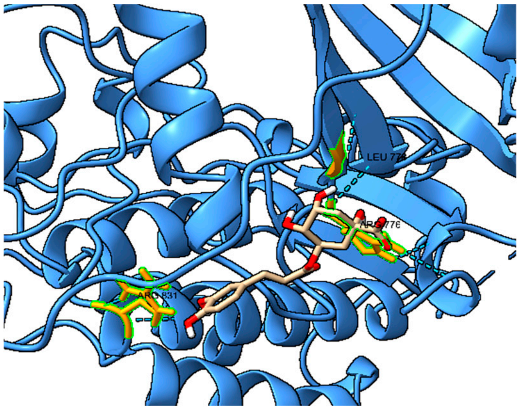

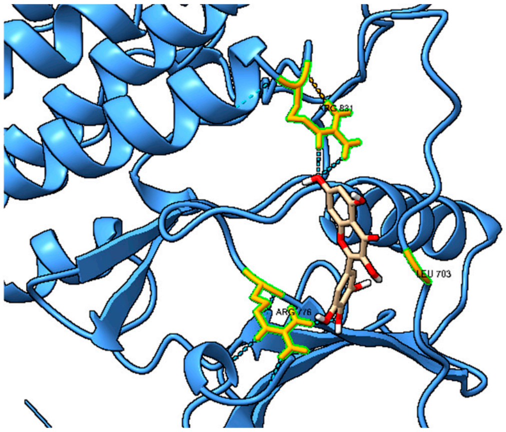

(S1a) **(S1b)**

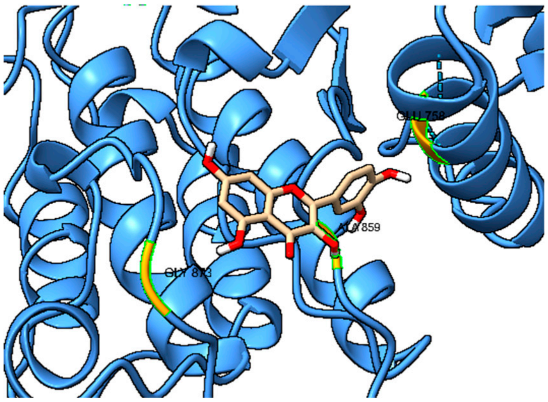

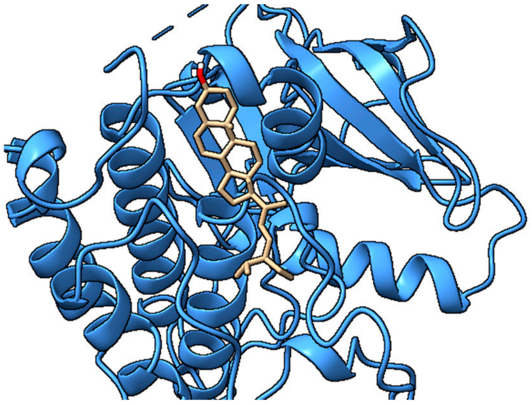

(S1c) **(S1d)**

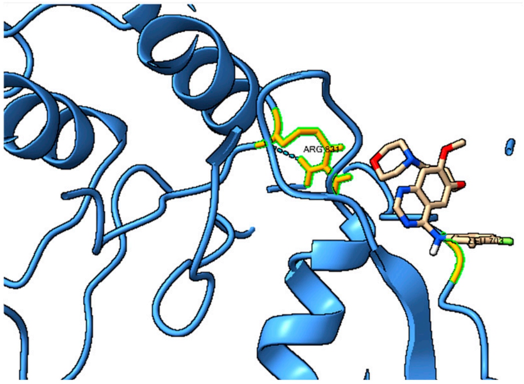

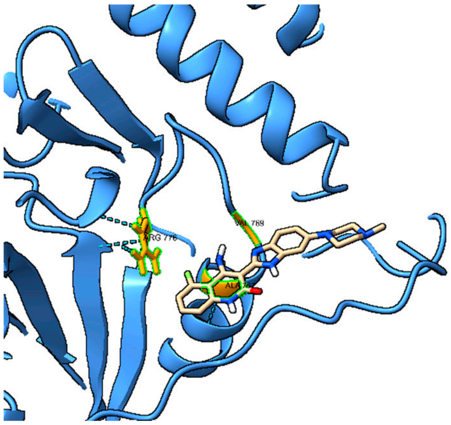

(S1e) **(S1f)**

Figure S1. 3D diagrams illustrating the orientation of ligands within the binding pocket of HER1. (S1a) 3-O-caffeoylquinic acid (CID: 1794427), (S1b) myricetin (CID: 5281672), (S1c) quercetin (CID: 5280343), (S1d) stigmasterol (CID: 5280794), (S1e) gefitinib (CID: 123631), and (S1f) dovitinib (CID: 135398510).

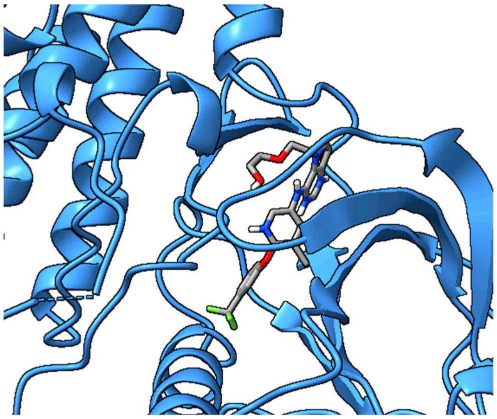

(S2a)

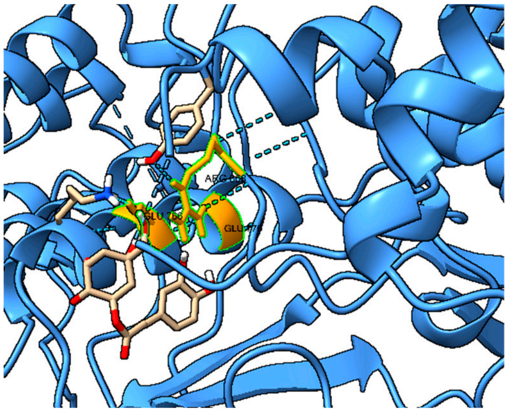

(S2b)

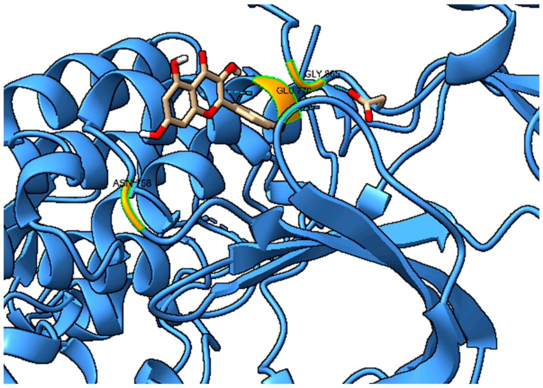

(S2c)

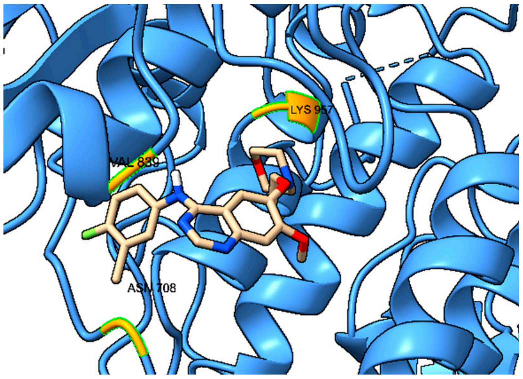

(S2d)

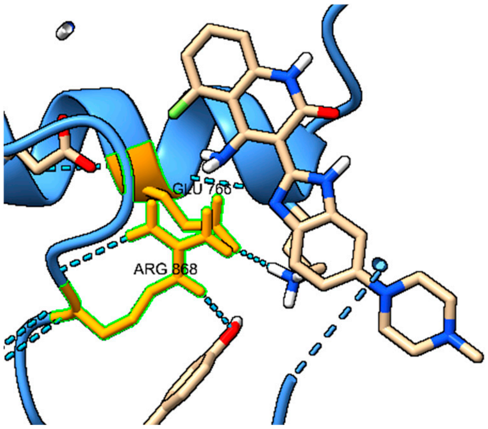

(S2e)

Figure S2. 3D diagrams illustrating the orientation of ligands within the binding pocket of HER2. (S2a) stigmasterol (CID: 5280794), (S2b) 3-O-caffeoylquinic acid (CID: 1794427), (S2c) kaempferol (CID: 5280863), (S2d) gefitinib (CID: 123631) and (S2e) dovitinib (CID: 135398510).

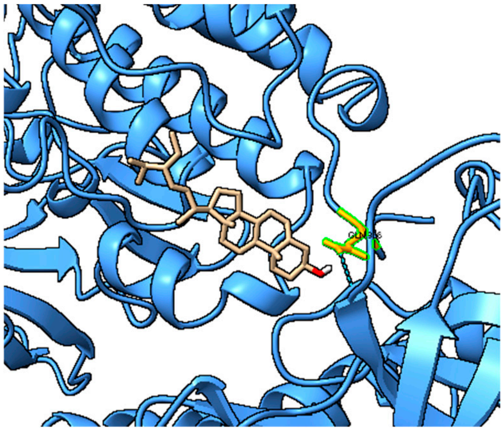

(S3a)

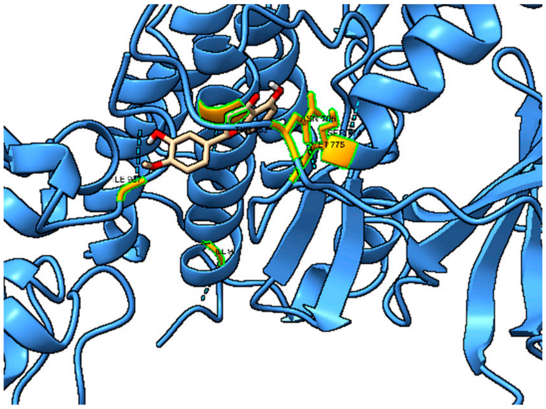

(S3b)

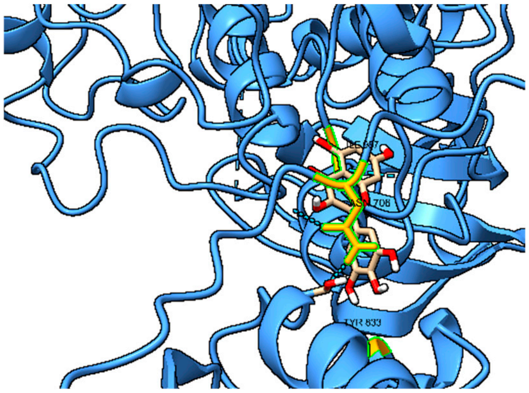

(S3c)

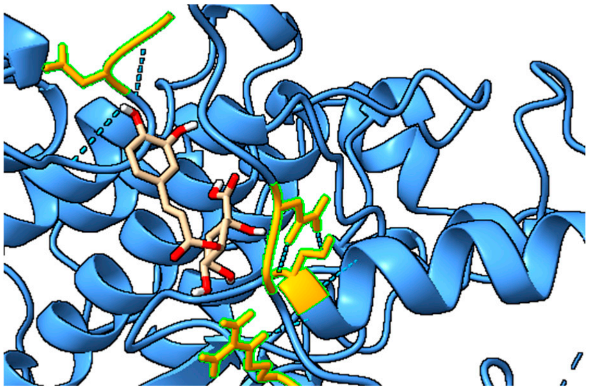

(S3d)

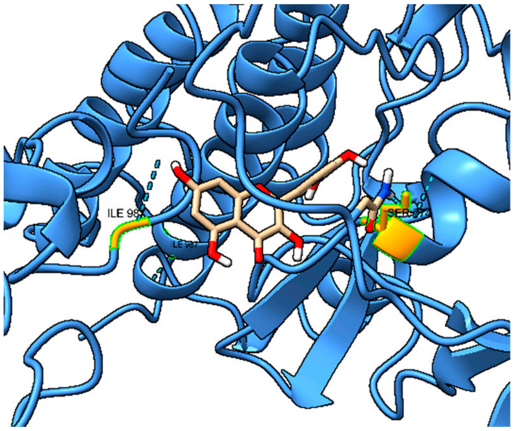

(S3e)

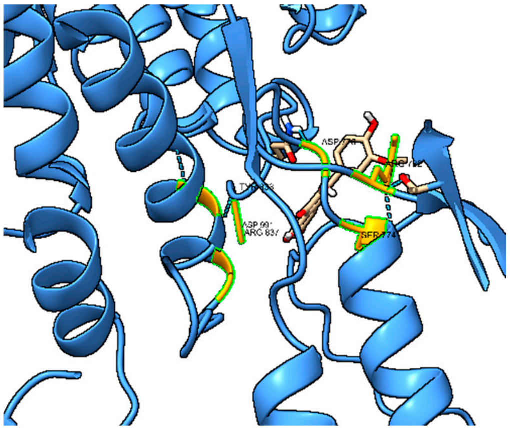

(S3f)

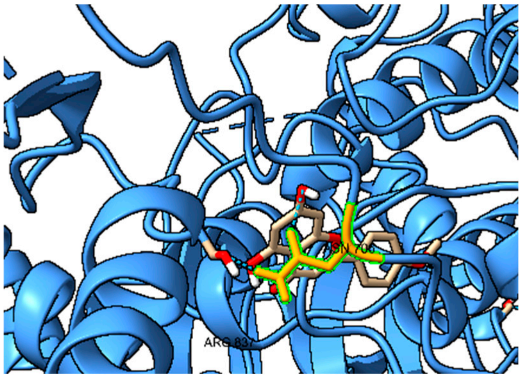

(S3g)

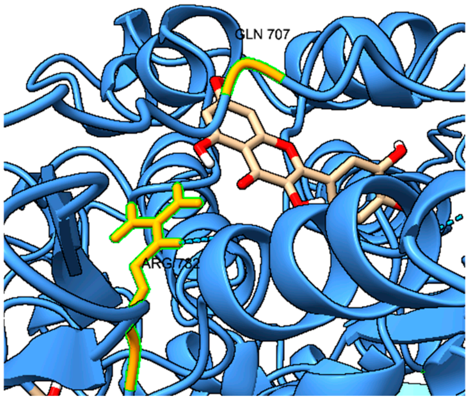

(S3h)

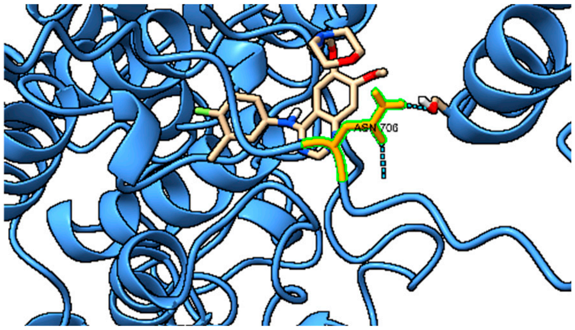

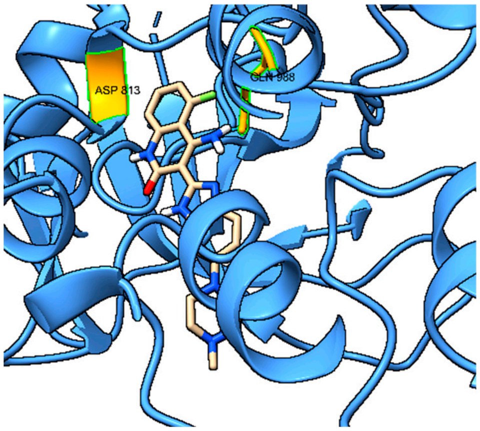

(S3i) **(S3j)**

Figure S3. 3D diagrams illustrating the orientation of ligands within the binding pocket of HER4. (S3a) stigmasterol (CID: 5280794), (S3b) hesperetin (CID: 72281), (S3c) myricetin (CID: 5281672), (S3d) 3-O-caffeoylquinic acid (CID: 1794427), (S3e) quercetin (CID: 5280343), (S3f) isorhamnetin (CID: 5281654), (S3g) acacetin (CID: 5280442), (S3h) rhamnetin (CID: 5281691), (S3i) gefitinib (CID: 123631) and (S3j) dovitinib (CID: 135398510).
