# Supplementary material for: Molecular Docking and Drug-Likeness of Salicornia-Derived Phytochemicals Against HER Receptors
Source: Curr Issues Mol Biol. 2025 Jun 27;47(7):495. doi: 10.3390/cimb47070495 (PMC12293734; doi:10.3390/cimb47070495)
Supplement: Supplementary file 1 [file cimb-47-00495-s001.zip › Supplementary Table S1.pdf]

Table S1. Comparison of docking score of proposed compounds with known active and inactive ligands for protocol validation.

S/N	Chemical ID	Name of the Chemical	Binding Energy (ΔG) (kcal/mol)	Type of the Compound
1	CID_ 6474310	3,5-di-O-caffeoylquinic acid	-8.7	Proposed (01)
2	CID_5328245	N-(3-bromophenyl) benzo[g]quinazolin-4-amine	-8.5	Active
3	CID_2428	N-(3-bromophenyl)-1H-imidazo[4,5-g] quinazolin-8-amine	-8.1	Active
4	CID_1794427	3-O-caffeoylquinic acid	-7.7	Proposed (02)
5	CID_10231458	N-(4-isopropoxyphenyl)-4-(6-methoxy-7-(3-morpholinopropoxy) quinazolin-4-yl) piperazine-1-carboxamide	-7.7	Inactive
6	CID_3038522	Tandutinib	-7.7	Inactive
7	CID_135431661	4-[4-[3-[4-[(propan-2-ylamino) methyl] anilino]-1H-pyrazol-5-yl] phenyl] benzene-1,3-diol	-7.6	Inactive
8	CID_5328753	Propanedinitrile, (2,3-dihydro-5,6-dihydroxy-1H-inden-1-ylidene)-	-6.9	Inactive
